# A dietary cholesterol challenge study to assess *Chlorella* supplementation in maintaining healthy lipid levels in adults: a double-blinded, randomized, placebo-controlled study

**DOI:** 10.1186/s12937-016-0174-9

**Published:** 2016-05-13

**Authors:** Sangmi Kim, Joohee Kim, Yeni Lim, You Jin Kim, Ji Yeon Kim, Oran Kwon

**Affiliations:** 1 Department of Nutritional Science and Food Management, Ewha Womans University, Seoul, 03760 Republic of Korea; 2 Department of Food Science and Technology, Seoul National University of Science and Technology, Seoul, 01811 Republic of Korea

**Keywords:** Carotenoids, *Chlorella*, Dietary cholesterol challenge, Healthy adult

## Abstract

**Background:**

Previous animal studies suggested that *Chlorella*, a unicellular green alga, has a preventive role in maintaining serum cholesterol levels against excess dietary cholesterol intake. This study aimed to conduct a pioneering investigation to clarify this issue in healthy subjects by adopting a dietary cholesterol challenge, which has not been used previously in similar studies of *Chlorella* in hypercholesterolemia.

**Methods:**

In this double blind, randomized, placebo-controlled study, 34 participants ingested 510 mg of dietary cholesterol from three eggs concomitantly with a usual dose of *Chlorella* (5 g/d) or a matched placebo for 4 weeks.

**Results:**

The dietary cholesterol challenge induced consistently higher concentrations of serum total cholesterol (TC, *P <* 0.001), LDL-C (*P =* 0.004), and HDL-C (*P* = 0.010) compared with baseline values, suggesting that the challenge was reliable. Thus, we observed a preventive action of *Chlorella* in maintaining serum TC versus placebo levels (3.5 % versus 9.8 %, respectively; *P* = 0.037) and LDL-C versus placebo levels (1.7 % versus 14.3 %, respectively; *P* = 0.012) against excessive dietary cholesterol intake and in augmenting HDL-C versus placebo levels (8.3 % versus 3.8 %, respectively). Furthermore, serum α-carotene showed the best separation between the placebo and *Chlorella* groups (R^2^X and R^2^Y > 0.5; Q^2^ > 0.4).

**Conclusion:**

The results suggest that a fully replicated dietary cholesterol challenge may be useful in assessing the effectiveness of dietary supplements in maintaining the serum lipid profiles of adults whose habitual diets are high in cholesterol.

**Trial registration:**

WHO International Clinical Trials Registry Platform (KCT0000258)

## Background

Cardiovascular disease (CVD) is the leading cause of mortality worldwide as a result of the aging population and increased urbanization. A high serum cholesterol level is thought to be a major risk factor for CVD [1]. Each 1 % increase in the serum cholesterol is predicted to increase the risk of coronary disease by approximately 2 % [2]. An impressive body of evidence has accumulated regarding the hypocholesterolemic effects associated with several plant-based foods compared with the placebo group in at risk subjects such as those with mild to severe hypercholesterolemia [3] or compared before and after administration in healthy subjects [4]. In animal models, a preventive effect of a food or food ingredient against hypercholesterolemia could be shown by the co-administration of excess dietary cholesterol in normal animals [5]. However, no successful placebo-controlled clinical trial has yet been explored to validate this concept in human subjects.

*Chlorella* is a unicellular green alga that contains a wide array of nutrients, including carotenoids, chlorophyll, minerals, vitamins and long-chain polyunsaturated fatty acids [5]. Animal experiments have shown that *Chlorella* inhibited the intestinal absorption of excess cholesterol from the diet and enhanced fecal steroid excretion, thereby preventing hypercholesterolemia [6]. However, several previous human clinical trials, including our own, have attempted only to determine only whether *Chlorella* supplementation is useful to reduce the high cholesterol levels of hypercholesterolemic subjects [7].

Here, we performed a double-blinded, randomized, placebo-controlled trial to investigate the hypothesis that a dietary cholesterol challenge might be useful to evaluate the effectiveness of *Chlorella* supplementation in maintaining healthy serum cholesterol concentration in adults consuming a high cholesterol meal. This approach is based on the previous findings of other investigations, which indicated that consuming three eggs daily could increase serum cholesterol levels in healthy subjects [8, 9]. Furthermore, we examined whether carotenoids derived from the eggs may have hampered the prediction of the responses of serum lipids to *Chlorella* carotenoids by using partial least squares (PLS) regression.

## Methods

### Test samples

Tablets containing 416 mg of dried *Chlorella* (*Chlorella vulgaris*) or a color-matched placebo (lactose) were kindly provided by Daesang Corp. (Seoul, Korea). *Chlorella* is a unicellular green alga and listed as a functional ingredient in the Korean Health/Functional Food Code with the following specifications: total chlorophyll content > 10 mg/g for identification; and lead < 3.0 mg/kg, cadmium < 1.0 mg/kg, total mercury < 0.5 mg/kg, total pheophorbide < 1,000 mg/kg, and coliform bacteria negative for purity. Daily *Chlorella* supplementation (12 tablets/d) provides 5 g of *Chlorella* containing the following nutrients: 3 g of protein, 180 mg of total carbohydrates, 640 mg of total lipids, 650 mg of dietary fiber, 225 mg of ash, 2,945 IU of vitamin A, 3.7 mg of vitamin C, and 1.1 mg of vitamin E [6]. The dietary source of the cholesterol challenge were pasteurized whole eggs were purchased from a retail market in Seoul, Korea. Daily egg consumption (3 eggs/d) provides 540 μg of lutein, 330 μg of zeaxanthin, 10 μg of β-carotene and trace amount of α-carotene, and 510 mg cholesterol.

### Study design

The double blind, randomized, and placebo-controlled study was performed in two phases, a 4-week lead-in phase and a 4-week intervention phase. The length of the intervention phase was determined based on a meta-analysis of plant sterols as cholesterol lowering agents [10]. To detect the postulated difference with 80 % power using a two-sided 5 %, and a 20 % dropout rate, the sample size was estimated to be 34 subjects. The study protocol was approved by the Institutional Review Boards of Ewha Womans University (Seoul, Korea) and registered at the WHO International Clinical Trials Registry Platform as KCT0000258. The study was conducted in accordance with the Declaration of Helsinki, and the results are reported according to the Consolidated Standards of Reporting Trials guidelines [11].

### Subjects

After the initial screening, 34 eligible subjects were entered at baseline. For study inclusion, a serum total cholesterol (TC) levels of < 5.18 mmol/L (desirable level) [12] was required of the subjects at the screening visit. The following exclusion criteria were applied: the regular use of medications or dietary supplements; presence of CVD, hypertension, type 2 diabetes, liver disease, renal failure, thyroid disease, or malignant tumors; a family history of hypercholesterolemia; known hypersensitivity to the study products; and pregnancy or lactation. All of the participants provided written informed consent before participation.

### Study procedures

Following the 4-week lead-in period, the participants were randomly assigned to either the placebo or *Chlorella* group. At the beginning of the study, the participants received one bottle of the test sample and 21 eggs (packaged in threes for a daily supply), enough for seven days, thus allowing for weekly visits. They were asked to take four tablets of the test sample and one egg with each meal, for a total of 5 g of *Chlorella* powder and 510 mg of cholesterol daily. Cooking methods for the eggs were provided to thereby minimize influences on carotenoid absorption. To determine a compliance status, the subjects were asked to answer whether they had followed the dietary instruction and the remaining pills were counted at each visit. The subjects were also required to report any possible adverse events. To assess nutrient intake and monitor dietary compliance, three-day dietary records were analyzed using a computer aided nutritional analysis program (Can-Pro 3.0, the Korean Nutrition Society, Seoul, Korea).

### Outcome measurements

At the beginning of the study and 4 weeks after the intervention, overnight fasting blood was collected in serum separator tubes (Becton Dickinson, Franklin Lakes, NJ, USA). The samples were then centrifuged, and the supernatants were stored at -80 °C before analysis. The serum TC, triglycerides (TG), and high-density lipoprotein cholesterol (HDL-C) concentrations were assessed using commercially available kits (Daiichi Pure Chemicals, Tokyo, Japan). The low-density lipoprotein cholesterol (LDL-C) level was calculated using the Friedewald equation (LDL-C = TC–HDL-C–TG/2.2). Serum carotenoid concentrations were measured by HPLC (Shiseido SP 3023, Tokyo, Japan) with a reverse-phase C18 column (Shiseido Capcell Pak, MG type, 5 μm, 4.6 × 250 mm) and UV detector.

### Statistical analysis

Data analyses were performed on the intention-to-treat (ITT) population, which included all randomized subjects. Additionally, per protocol (PP) analysis was performed on participants who completed the study. The results of the ITT and PP analyses were comparable, thus only those of the ITT analysis are shown. Variables were tested for normal distribution using the Shapiro-Wilk *W*-test and log transformation was performed on skewed variables. A Student’s *t*-test or Fisher’s exact test was used to compare the baseline characteristics between groups. An analysis of variance (ANOVA) mixed-effects model was applied to compare changes in nutrient intake, serum lipids, and serum carotenoids with group and time as fixed effects factors and subject as random effect factor. A two-tailed value of *P* < 0.05 was considered to be significantly different. PLS regression was used to correlate measured values of serum carotenoids for serum lipids and identify predictors for responses to *Chlorella* during the dietary cholesterol challenge. PLS regression with 5-fold cross-validation leads to the calculation of the R^2^X, R^2^Y, and Q^2^ factors, representing the explanation, fitness, and prediction power, respectively. *R* ^2^ > 0.5 and Q^2^ > 0.4 [13] were considered acceptable for PLS regression. The data were analyzed using the SAS 9.3 statistical software (SAS Institute, Cary, NC, USA) or TANAGRA (version1.4.50, University of Lyon, France).

## Results

### Subject characteristics and dietary monitoring

**Fig. 1:**
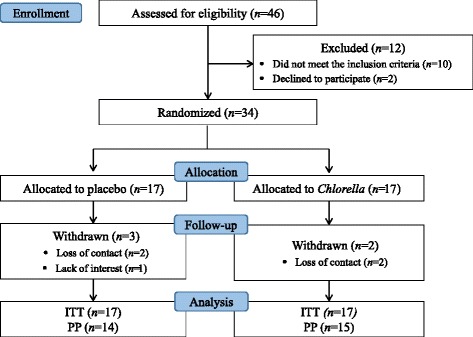
CONSORT diagram for the flow of subjects through the study. ITT, intention-to-treat; PP, per protocol

The CONSORT flow chart of the study is shown in Fig. 1. Following the 4-week lead-in period, 34 eligible subjects were randomized into two groups. Five subjects were withdrawn from the study due to lack of interest (*n* = 1) or loss of contact (*n* = 4). The compliance was excellent (mean compliance ratio for all subjects = 97.5 %) with no subjects were excluded due to insufficient compliance. No participant reported any significant subjective symptoms or serious events.

The participant characteristics at baseline are shown in Table 1. All subjects were in good health, as determined by physical examination and routine blood and urine biochemical screening. Randomization was successful, and no significant differences were observed in the baseline findings between the groups. The daily intakes of calories and some selected nutrients at baseline and week 4 are listed in Table 2. Within each group, cholesterol intake increased by approximately 3-fold by adding three eggs to the usual diet (*P* < 0.001 for time effect). No significant between-group differences were observed for any nutrient, however, β-carotene intake was significantly increased in the *Chlorella* group compared to the placebo group (*P* = 0.010 for interaction). Unexpectedly, carbohydrate intake was decreased in both groups (*P* = 0.033 for time effect).

**Table 1:** Characteristics of the participants at baseline

	Placebo group (*n* = 17)	*Chlorella* group (*n* = 17)	*P*-value
Age, y	23.7 ± 0.8	23.2 ± 0.6	0.639
Female/Male, *n*	15/2	15/2	1.000
BMI, kg/m^2^	19.7 ± 0.5	20.7 ± 0.4	0.130
SBP, mmHg	106.4 ± 2.4	104.4 ± 2.3	0.557
DBP, mmHg	63.8 ± 1.9	64.6 ± 1.7	0.737
Fasting serum levels, mmol/L			
TC	4.24 ± 0.16	4.38 ± 0.12	0.495
TG	0.76 ± 0.07	0.83 ± 0.08	0.678
LDL-C	2.28 ± 0.11	2.49 ± 0.11	0.186
HDL-C	1.58 ± 0.08	1.55 ± 0.06	0.737

Values are the means ± SEMs (all such values). *P*-values are for Student’s *t*-test or Fisher’s exact test used to compare the between-group differences. *BMI* body mass index, *SBP* systolic blood pressure, *DBP* diastolic blood pressure, *TC* total cholesterol, *TG* triglycerides, *LDL-C* low-density lipoprotein cholesterol, *HDL-C* high-density lipoprotein cholesterol

**Table 2:** Mean daily intakes of selected nutrients at baseline and during the 4-week intervention

Nutrients	Placebo group (*n* = 17)		*Chlorella* group (*n* = 17)		*P*-value^a^		
	Week 0	Week 4	Week 0	Week 4	Group	Time	Group x time
Energy, kcal/d	1558 ± 124	1505 ± 92	1511 ± 111	1485 ± 81	0.800	0.642	0.860
Carbohydrate, g/d	204.0 ± 16.6	185.6 ± 13.7	214.7 ± 15.3	186.0 ± 11.7	0.734	0.033	0.680
Protein, g/d	63.0 ± 5.5	65.3 ± 3.2	56.2 ± 7.3	64.9 ± 3.8	0.400	0.065	0.394
Fat, g/d	52.7 ± 5.1	55.3 ± 3.3	46.2 ± 3.5	50.5 ± 2.5	0.189	0.338	0.815
SFA, *g/d*	8.2 ± 1.1	9.5 ± 1.1	7.4 ± 1.2	9.6 ± 0.8	0.723	0.071	0.643
PUFA, *g/d*	5.8 ± 0.7	6.5 ± 0.8	6.4 ± 0.9	6.4 ± 0.5	0.664	0.194	0.810
Cholesterol, mg/d	323.5 ± 54.5	725.9 ± 17.5	219.1 ± 32.2	754.1 ± 25.7	0.312	< 0.001	0.220
Dietary fibers, g/d	12.7 ± 1.3	12.6 ± 1.1	11.3 ± 1.1	11.9 ± 0.6	0.665	0.316	0.568
β-carotene, mg/d	2.2 ± 0.4	1.7 ± 0.3	1.9 ± 0.4	2.6 ± 0.2	0.266	0.302	0.010

Values are the means ± SEMs. Intakes were estimated from 3-day dietary records using CAN-pro (Korean Nutrition Society, Seoul, Korea). Egg, *Chlorella* and placebo intakes were included in the analysis. *SFA* saturated fatty acids, *PUFA* polyunsaturated fatty acids

^a^Mixed-effects ANOVA model, with group and time as fixed effects factors and subject as random effect factor

### The role of Chlorella in maintaining health serum lipid profiles following a dietary cholesterol challenge

No difference in baseline concentrations of serum lipids was observed between the two groups. After 4 weeks, the 3-egg dietary cholesterol challenge induced significant increases in serum TC (*P <* 0.001 for time effect), LDL-C (*P =* 0.004 for time effect), and HDL-C (*P* = 0.010 for time effect) in the placebo group. In contrast, concomitant ingestion of 5 g *Chlorella* significantly suppressed the increases of TC (9.8 % versus 3.5 %; *P* = 0.037 for interaction) and LDL-C (14.3 % versus 1.7 %; *P* = 0.012 for interaction), but accelerated the increase of HDL-C (3.8 % versus 8.3 %), a finding that lacked statistical significance (Table 3, upper part).

**Table 3:** Serum lipid and carotenoid responses to the 4-week intervention with *Chlorella* supplementation with three eggs daily

Fasting serum levels (mmol/L)	Placebo group (*n* = 17)		*Chlorella* group (*n* = 17)		*P*-value^a^		
	Week 0	Week 4	Week 0	Week 4	*Group*	*Time*	*Group x Time*
Serum lipids							
TC	4.24 ± 0.16	4.71 ± 0.15	4.38 ± 0.12	4.47 ± 0.12	0.962	< 0.001	0.037
LDL-C	2.28 ± 0.11	2.59 ± 0.11	2.49 ± 0.11	2.51 ± 0.12	0.619	0.004	0.012
HDL-C	1.58 ± 0.08	1.67 ± 0.10	1.55 ± 0.06	1.62 ± 0.05	0.917	0.010	0.404
Serum carotenoids							
Lutein	0.54 ± 0.05	0.89 ± 0.08	0.65 ± 0.03	1.39 ± 0.12	0.001	< 0.001	0.006
α-carotene	0.06 ± 0.01	0.07 ± 0.01	0.08 ± 0.01	0.24 ± 0.02	< 0.001	< 0.001	< 0.001
β-carotene	0.19 ± 0.02	0.27 ± 0.05	0.22 ± 0.02	0.29 ± 0.03	0.292	< 0.001	0.753

Values are the means ± SEMs. *TC* total cholesterol, *LDL-C* low-density lipoprotein cholesterol, *HDL-C* high-density lipoprotein cholesterol

^a^Mixed-effects ANOVA model with group and time as fixed effects factors and subject as random effect factor

### Serum carotenoids as a predictor of serum lipid responses to Chlorella supplementation

The daily ingestion of three eggs resulted in significant increases in the serum lutein, α-carotene and β-carotene levels (*P* < 0.001 for time effect for all) in the placebo group. Concomitant intake of *Chlorella* supplementation led to a significant enhancement of lutein (76 % versus 123 %; *P* = 0.006 for interaction) and α-carotene (31.2 % versus 228 %, *P* < 0.001 for interaction) levels. However, serum β-carotene concentration remained unchanged between the two groups (Table 3, lower part).

**Fig. 2:**
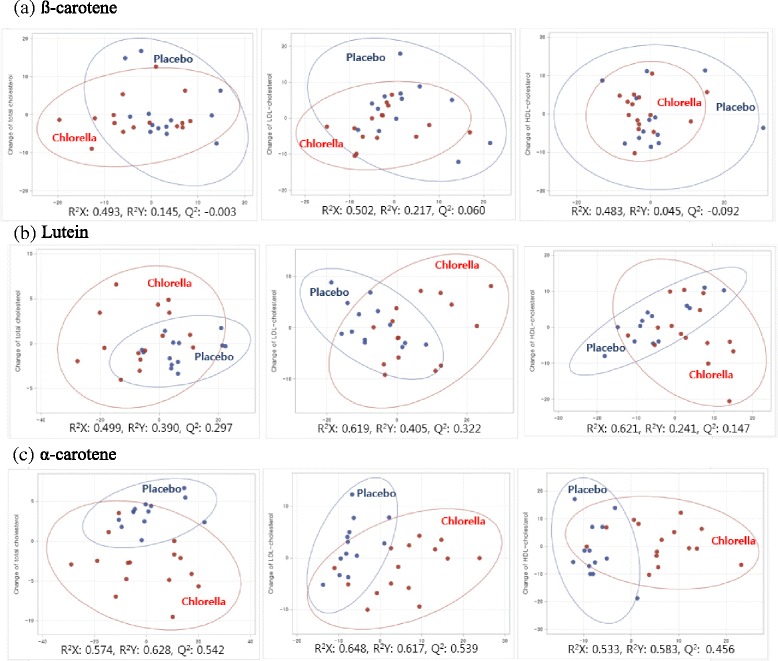
PLS-regression score plots of each serum lipid and carotenoid obtained from the placebo and *Chlorella* group. R^2^X, modeled variation of X; R^2^Y, predicted variation of Y; Q^2^, cross-validated prediction of Y. (**a**) β-carotene, (**b**) Lutein, (**c**) α-carotene

PLS regressions for each combination of serum carotenoids and lipids showed that data points from β-carotene (Fig. 2a) and lutein (Fig. 2b) were randomly dispersed in the plane and did not show clear discrimination between groups. In contrast, α-carotene showed better separations between the placebo and *Chlorella* groups (R^2^X and R^2^Y > 0.5; Q^2^ > 0.4) (Fig. 2c). The finding suggests that α-carotene may serve an important parameter for predicting the maintenance effect of *Chlorella* on healthy cholesterol concentrations.

## Discussion

The data obtained in this study validated the hypothesis that a dietary cholesterol challenge permitted the determination of a beneficial impact of *Chlorella* on modifying serum cholesterol concentration in subjects whose habitual diets are high in cholesterol. Early changes in metabolic markers may not be easily identified due to the robustness of homeostatic vigor of healthy subjects [14]. The administration of additional dietary cholesterol from three eggs daily created a temporary perturbation in serum cholesterol levels, thus providing a unique opportunity to evaluate the intervention effect of *Chlorella* on the earliest changes in serum lipid profiles. To the best of our knowledge, this is the first attempt to use a dietary cholesterol challenge to investigate the modifying effect of a food or food component on serum lipid profile in healthy subjects.

Notably, the serum lipid profile changes that ensued from the three egg consumption were modest; (1) the alterations were quantitatively within the normal ranges and the LDL particle subclass was identified as the larger LDL particle [15], which is believed to have a reduced susceptibility to oxidation and endothelial penetration [16]. Furthermore, the 0.40 mmol/L increase in the serum cholesterol we observed in this study in response to the ingestion of 510 mg of additional cholesterol from three eggs was in agreement with previous studies that estimated a 0.05 ~ 0.12 mmol/L increase in the serum TC for each additional 100 mg of dietary cholesterol [17, 18]. Together, these results suggest that the 4-week dietary cholesterol challenge from three eggs each day was fully replicated in magnitude the expected changes in serum cholesterol.

*Chlorella* contains a wide range of bioactive substances that may act to optimize lipid metabolism. In our previous study, we observed that *Chlorella* carotenoids are highly bioavailable [7, 19]. In this study, we examined whether serum carotenoids derived from *Chlorella* may be used to predict serum lipid responses to *Chlorella* consumption during a dietary cholesterol challenge. Because the eggs also contain high level of carotenoids, we first compared the serum carotenoid levels for each group. In the case of lutein, the daily lutein consumption from three eggs (540 μg/d) increased mean serum lutein levels by 58 % at 4 weeks compared to the baseline value. When *Chlorella* lutein (7,450 μg/d) was added, the mean serum lutein levels increased 2-fold compared to egg consumption only. In regard to α-carotene, *Chlorella* provided high levels (500 μg/d), whereas eggs provided trace amounts. The mean serum α-carotene level was increased 8-fold following *Chlorella* supplementation compared to egg consumption only. However, the additional β-carotene consumption from *Chlorella* (900 μg/d) was not associated with a significant increase of serum β-carotene level compared to egg consumption only, likely because of the rapid conversion of β-carotene to vitamin A [20].

Then, PLS regression analysis was performed to identify the carotenoids that contributed to predicting the effectiveness of *Chlorella* on serum lipid profile during the dietary cholesterol challenge. Consistent with our previous study in subjects with hypercholesterolemia, the data points from α-carotene showed a better separation by PLS regression analysis. Taken together, the cumulative data suggested that serum α-carotene concentration might be useful, at least in part, to predict *Chlorella* effects on serum lipid concentrations during the dietary cholesterol challenge. However, it is a limitation of this study that we did not determine the underlying mechanism that could explain how the results obtained may have been due to the high concentration of serum α-carotene. Future studies will be needed to confirm the concomitant effects of carotenoids on cholesterol absorption and to understand the pathways and responses initiated by *Chlorella* supplementation following a high dietary cholesterol challenge in laboratory animals and human subjects.

## Conclusions

Given the modest nature of the serum lipid response to dietary cholesterol challenge from three eggs daily for 4 weeks, our findings provide an essential foundation for applying a dietary cholesterol challenge can be applied to evaluate candidate foods or food components with hypocholesterolemic properties in healthy subjects. Thereby it is plausible to implicate that *Chlorella* as playing a useful role in maintaining healthy serum cholesterol levels in the environment of free access to high-lipid foods, thus preventing or delaying the risk for CVD.

## Abbreviations

CVD: cardiovascular disease
HDL-C: high-density lipoprotein cholesterol
HPLC: high performance liquid chromatography
ITT: intention-to-treat
LDL-C: low-density lipoprotein cholesterol
PP: per protocol
TC: total cholesterol
TG: triglycerides

## References

[1] Wu T, Trevisan M, Genco RJ, Falkner KL, Dorn JP, Sempos CT (2000). Examination of the relation between periodontal health status and cardiovascular risk factors: serum total and high density lipoprotein cholesterol, C-reactive protein, and plasma fibrinogen. Am J Epidemiol.

[2] The Lipid Research Clinics Coronary Primary Prevention Trial results. II. The relationship of reduction in incidence of coronary heart disease to cholesterol lowering. JAMA 1984; 251:365-374. 6361300

[3] Lu TM, Chiu HF, Shen YC, Chung CC, Venkatakrishnan K, Wang CK (2015). Hypocholesterolemic efficacy of quercetin rich onion juice in healthy mild hypercholesterolemic adults: a pilot study. Plant Foods Hum Nutr.

[4] Alvarez-Suarez JM, Giampieri F, Tulipani S, Casoli T, Di Stefano G, Gonzalez-Paramas AM, Santos-Buelga C, Busco F, Quiles JL, Cordero MD (2014). One-month strawberry-rich anthocyanin supplementation ameliorates cardiovascular risk, oxidative stress markers and platelet activation in humans. J Nutr Biochem.

[5] Bocanegra A, Bastida S, Benedí J, Ródenas S, Sánchez-Muniz FJ (2009). Characteristics and nutritional and cardiovascular-health properties of seaweeds. J Med Food.

[6] Lee HS, Park HJ, Kim MK (2008). Effect of Chlorella vulgaris on lipid metabolism in Wistar rats fed high fat diet. Nutr Res Pract.

[7] Ryu NH, Lim Y, Park JE, Kim J, Kim JY, Kwon SW, Kwon O (2014). Impact of daily Chlorella consumption on serum lipid and carotenoid profiles in mildly hypercholesterolemic adults: a double-blinded, randomized, placebo-controlled study. Nutr J.

[8] Weggemans RM, Zock PL, Katan MB (2001). Dietary cholesterol from eggs increases the ratio of total cholesterol to high-density lipoprotein cholesterol in humans: a meta-analysis. Am J Clin Nutr.

[9] Fernandez ML (2006). Dietary cholesterol provided by eggs and plasma lipoproteins in healthy populations. Curr Opin Clin Nutr Metab Care.

[10] Abumweis SS, Barake R, Jones PJ: Plant sterols/stanols as cholesterol lowering agents: A meta-analysis of randomized controlled trials. Food Nutr Res 2008, 52 doi: 10.3402/fnr.v52i0.1811 10.3402/fnr.v52i0.1811 PMC2596710 19109655

[11] Moher D, Hopewell S, Schulz KF, Montori V, Gøtzsche PC, Devereaux PJ, Elbourne D, Egger M, Altman DG, CONSORT (2012). CONSORT 2010 explanation and elaboration: updated guidelines for reporting parallel group randomised trials. Int J Surg.

[12] National Cholesterol Education Program (NCEP) Expert Panel on Detection, Evaluation, and Treatment of High Blood Cholesterol in Adults (Adult Treatment Panel III) (2002). Third report of the National Cholesterol Education Program (NCEP) expert panel on detection, evaluation, and treatment of high blood cholesterol in adults (adult treatment panel III) final report. Circulation.

[13] Paban V, Fauvelle F, Alescio-Lautier B (2010). Age-related changes in metabolic profiles of rat hippocampus and cortices. Eur J Neurosci.

[14] Wopereis S, Rubingh CM, van Erk MJ, Verheij ER, van Vliet T, Cnubben NH, Smilde AK, van der Greef J, van Ommen B, Hendriks HF (2009). Metabolic profiling of the response to an oral glucose tolerance test detects subtle metabolic changes. PLoS One.

[15] Greene CM, Zern TL, Wood RJ, Shrestha S, Aggarwal D, Sharman MJ, Volek JS, Fernandez ML. Maintenance of the LDL cholesterol: HDL cholesterol ratio in an elderly population given a dietary cholesterol challenge. J Nutr. 2005;135:2793–8. 10.1093/jn/135.12.2793 16317122

[16] Aiyer AN, Kip KE, Marroquin OC, Mulukutla SR, Edmundowicz D, Reis SE (2007). Racial differences in coronary artery calcification are not attributed to differences in lipoprotein particle sizes: the Heart Strategies Concentrating on Risk Evaluation (Heart SCORE) study. Am Heart J.

[17] Hegsted DM (1986). Serum-cholesterol response to dietary cholesterol: a re-evaluation. Am J Clin Nutr.

[18] Hopkins PN (1992). Effects of dietary cholesterol on serum cholesterol: a meta-analysis and review. Am J Clin Nutr.

[19] Jung HY, Ok HM, Park MY, Kim JY, Kwon O. Bioavailability of carotenoids from chlorella powder in healthy subjects: A comparison with marigold petal extract. J Funct Foods. 2016;21:27–35.

[20] Yeum KJ, Russell RM (2002). Carotenoid bioavailability and bioconversion. Annu Rev Nutr.

